# Di(2-ethylhexyl) phthalate-induced apoptosis in rat INS-1 cells is dependent on activation of endoplasmic reticulum stress and suppression of antioxidant protection

**DOI:** 10.1111/jcmm.12409

**Published:** 2014-11-23

**Authors:** Xia Sun, Yi Lin, Qiansheng Huang, Junpeng Shi, Ling Qiu, Mei Kang, Yajie Chen, Chao Fang, Ting Ye, Sijun Dong

**Affiliations:** Key Lab of Urban Environment and Health, Institute of Urban Environment, Chinese Academy of SciencesXiamen, China

**Keywords:** apoptosis, β cells, Di(2-ethylhexyl) phthalate, endoplasmic reticulum stress, oxidative damage

## Abstract

Di(2-ethylhexyl) phthalate (DEHP) is used as plasticizer and is ubiquitously found in the environment. Exposure to DEHP has been linked to an increased incidence of type 2 diabetes. Pancreatic β-cell dysfunction is a hallmark of type 2 diabetes; however, it is unknown whether DEHP exposure contributes to this risk. Here, we aimed to investigate the cytotoxic effects of DEHP on INS-1 cells and to further explore the related underlying mechanisms. INS-1 cells were exposed to 0, 5, 25, 125 or 625 μM DEHP for 24 hrs. Cell viability, glucose-stimulated insulin secretion, reactive oxygen species (ROS) generation, cellular antioxidant response, Ca^2+^ homoeostasis and the levels of genes and proteins involved in endoplasmic reticulum (ER) stress were measured. The results showed that DEHP decreased insulin secretion and content and induced apoptosis in INS-1 cells in a dose-dependent manner. Furthermore, ROS generation was increased and Nrf2-dependent antioxidant defence protection was dysregulated in INS-1 cells after DEHP exposure. Most importantly, DEHP effectively depleted ER Ca^2+^ and triggered the ER stress response as demonstrated by the elevated transcription and translation of the ER chaperone GRP78 and GRP94, the increased phosphorylation of protein kinase R-like endoplasmic reticulum kinase (PERK) and its downstream substrate eukaryotic translation initiation factor 2α (eIF2α), as well as the increased levels of activating transcription factor 4 (ATF4) and C/EBP homologous protein (CHOP). Taken together, DEHP exerted toxic effects on INS-1 cells by inducing apoptosis, which is dependent on the activation of the PERK–ATF4–CHOP ER stress signalling pathway and the suppression of Nrf2-dependent antioxidant protection.

## Introduction

Di (2-ethyl hexyl) phthalate (DEHP) is one of various ubiquitous environmental endocrine disruptors widely used as plasticizers or solvents in food packaging, medical devices, pharmaceutical formulations, household products and industrial plastic, *etc*. 1,2. DEHP is not covalently bound to the plastic matrix; therefore it can easily leach out of products into the environment. Human exposure to DEHP is widespread and frequent. Biomonitoring data continuously collected in the National Health and Nutrition Examination Survey (NHANES) since 1999 showed that a majority of individuals (>6 years old) in the United States had detectable concentrations of DEHP and other metabolites in the urine 3. In Canada, a national health measure survey (CHMS) from 2007 to 2009 also indicated that DEHP metabolites were detected in >90% of Canadians 4.

Recently, an increasing number of studies have provided evidence of a significant association between DEHP exposure and adverse glucometabolic outcome, including insulin resistance or type 2 diabetes. In a cross-sectional study of a representative sample of U.S. men, urinary concentrations of DEHP metabolites were first found to correlate with increased insulin resistance 5. A correlation was also found between the level of DEHP metabolites and the occurrence of type 2 diabetes among Mexican women 6. A more recent study performed in 2350 women between 20 and 79 years of age who participated in the 2001–2008 NHANES further revealed a strong correlation between the urinary level of DEHP and the occurrence of diabetes in the United State 7. In animal studies, DEHP was also associated with diabetogenic effects. For example, administration of 10 or 100 mg/kg/day DEHP for 30 days significantly disrupted insulin signal transduction and diminished plasma membrane GLUT4 level in both adipose and skeletal muscle of adult male albino rats, implying that DEHP was able to induce insulin resistance by decreasing glucose oxidation and uptake in peripheral tissue 8,9.

In addition to the increased insulin resistance, progressive pancreatic β-cell dysfunction is a major pathogenic component in type 2 diabetes 10. β cells express extremely low levels of antioxidant enzymes such as catalase and glutathione peroxidase 11,12, and they are particularly susceptible to reactive oxygen species (ROS)-elicited oxidative stress damage. DEHP has been reported to be involved in peroxisome proliferator-activated receptor activation; therefore, it may induce oxidative stress by increasing peroxidase expression and ROS generation 13. Indeed, NHANES in 1999–2006 reported that higher urinary concentrations of DEHP metabolites were inversely associated with bilirubin 14, a marker of oxidative stress in the prediction of metabolic disease because of its antioxidant activity 15. Furthermore, animal studies confirmed that DEHP exposure causes oxidative stress in reproductive tissues and cells by disrupting antioxidant defences and increasing ROS 16,17. Oxidative stress is mostly implicated as a potential mechanism for functional failure of pancreatic β cells in type 2 diabetes. Whether exposure to DEHP impairs β cells through an oxidative stress pathway remains to be elucidated.

Reactive oxygen species is a group of upstream signalling molecules in the endoplasmic reticulum (ER) stress signalling pathway 18,19. In pancreatic β cells, ER is a crucial site for insulin biosynthesis 20. ER stress-mediated β-cell dysfunction plays an important role in type 2 diabetes. In a previous study, Wistar rat offspring perinatally exposed to DEHP at 1.25 and 6.25 mg/kg/day showed β-cell defects that was characterized by hypertrophic rough ER, reduced mass and decreased insulin content as well as altered the expression of ER stress genes in islets 21. ER damage could be another important mechanism underlying DEHP-induced β-cell dysfunction.

In the present study, we treated cells lines derived from a rat insulinoma, INS-1 cells, with DEHP and assessed cellular insulin secretion and content, ROS generation and antioxidant response, as well as activation of the ER stress pathway and apoptosis. This study is the first to identify the potential cytotoxicity of DEHP on β cells and to evaluate the molecular mechanism involved in DEHP-induced β-cell dysfunction and apoptosis.

## Materials and methods

### Cell culture and treatment

INS-1 cells (between passages 38 and 67), were purchased from Biohermes (Shanghai, China) and were cultured in RPMI-1640 medium supplemented with 10% foetal bovine serum (FBS), 50 μM β-mercaptoethanol, 25 mM HEPES, 2 mM L-glutamine, 5.6 mM glucose, 100 U/ml penicillin and 100 mg/ml streptomycin at 37°C under 5% CO_2_. Culture medium, FBS and supplements were purchased from Invitrogen (Carlsbad, CA, USA). The cells were seeded into 96-, 24- or 6-well plates depending on the experiment. After a 24-hr pre-culture, the cells were incubated with serial concentrations of DEHP (0, 5, 25, 125 or 625 μM) for 24 hrs. DEHP was dissolved in dimethyl sulfoxide (DMSO); the final concentration of DMSO in medium was 0.1% (v/v) in all experiments.

### Glucose-stimulated insulin secretion (GSIS)

INS-1 cells were cultured in 24-well plates and exposed to DEHP for 24 hrs. After exposure, the medium was removed and the cells were incubated with Krebs–Ringer bicarbonate HEPES buffer (KRBH) supplemented with 0.5% BSA and 3.0 mM glucose for 1 hr. Afterwards, the cells were challenged to secrete insulin in KRBH buffer containing 3.0, 5.6 or 16.7 mM glucose for an additional 1 hr. Supernatants were collected and measured with a Rat Insulin ELISA kit (Millipore, Billerica, MA, USA).

### Cell viability assay

INS-1 cells were seeded into 96-well plates and stimulated with DEHP for 24 hrs. Then, the medium was removed and the cells were incubated with 0.5 mg/ml MTT (Sigma-Aldrich, St. Louis, MO, USA) for 4 hrs at 37°C. The resulting formazan crystals were solubilized by DMSO and the absorbance was read at 490 nm on a SpectraMAX M5 microplate reader (Molecular Devices, Sunnyvale, CA, USA). The OD values of controls were taken set at 100% and the values for DEHP-treated cells were expressed as % of controls.

### Cell proliferation assay

Cell proliferation was assessed with a BrdU ELISA kit (Roche, Mannheim, Germany) in accordance with the manufacturer's instruction. After a 24-hr treatment, 10 μM BrdU labelling medium was added to the cells for 2 hrs in a 37°C incubator. Then, cells were fixed with FixDenat and incubated with anti-BrdU-POD for 90 min. at room temperature. Finally, the peroxidase substrate solution was added, and the cells were incubated for further 30 min. at room temperature. The chemiluminescence intensity was obtained by measuring the value at 370 nm with a reference at 492 nm using a microplate reader.

### Apoptosis detection

The Annexin V-FLUOS Staining Kit (Roche) was used to detect cellular apoptosis. Briefly, cells were seeded in 6-well plates and treated with DEHP for 24 hrs. Then, the cells were harvested by trypsin, washed twice with pre-cooled PBS, and re-suspended in 100 μl of Annexin V-Fluos binding solution (5 mM CaCl_2_, 140 mM NaCl and 10 mM NaOH/HEPES, pH 7.4) containing 1:50 dilution of Annexin V–FITC conjugate and 1 μg/ml propidium iodide. After a 15 min. incubation, the cells were washed with binding buffer and the samples were analysed with a Quanta SC flow cytometer (Beckman Coulter, Brea, CA, USA).

### Determination of ROS production

Intracellular ROS generation was measured using a peroxide-sensitive fluorescent probe: 2,7-dichlorofluorescein diacetate (DCFH-DA; Sigma-Aldrich). In brief, cells were seeded in 6-well plates and exposed to various concentrations of DEHP for 24 hrs. Then, the cells were washed with PBS and co-incubated with 10 μM DCFH-DA for 30 min. at 37°C. After incubation, the cells were washed and re-suspended with ice-cold PBS. Intracellular ROS was imaged by the LSM 710 Laser Scanning Confocal Microscope (Zeiss, Jena, Germany) using a condition of 488 nm for excitation and 525 nm for emission. The image density was quantified with an Image Pro Plus version 6.0 software (Media Cybernetics, Bethesda, MD, USA). Five images per treatment were captured: one image in each of the four quadrants and one in the centre of the well 22. Equivalent adjustments for brightness and contrast were applied to each image.

### Immunofluorescence analysis of Nrf2 localization

Cells were seeded in sterile glass slides and exposed to various concentrations of DEHP for 24 hrs. After exposure, the cells were fixed with 4% paraformaldehyde solution for 15 min., and were then treated with permeation solution (0.2% Triton X-100 and 0.2% BSA in PBS) for 10 min. on ice. The cells were blocked with 0.02% Triton X-100 and 5% BSA in PBS for 2 hrs and incubated overnight with nuclear factor erythroid 2-related factor 2 primary antibodies (Nrf2, Q16236; 1:250; Epitomics, CA, USA) at 4°C. In the next day, the cells were incubated with Alexa Fluor® 555 Donkey Anti-Rabbit IgG (A31572; 1:500; Invitrogen) for 1.5 hrs. Finally, the slides were mounted with DAPI (1 μg/ml; Roche) for 10 min. and the subcellular localization of Nrf2 was imaged using an LSM 710 Laser Scanning Confocal Microscope.

### Intracellular Ca^2+^ concentration ([Ca2+]_i_) measurement

Intracellular Ca^2+^ concentrations [Ca^2+^]_i_ were measured using Ca^2+^-sensitive fluorescent Fluo-3/acetoxymethyl (AM; AAT Bioquest, Sunnyville, CA, USA). After 24 hrs of DEHP treatments, cells were incubated with Fluo-3/AM in Ca^2+^- and Mg^2+^-free HBSS (Invitrogen) at 37°C for 30 min. Then, the cells were washed with Ca^2+^- and Mg^2+^-free HBSS to remove the unloaded probes and incubated with the same saline solution supplemented with 1.0% (v/v) FBS at 37°C for 40 min. to completely de-esterify the dye. Finally, the cells were washed and re-suspended in Ca^2+^- and Mg^2+^-free HBSS until the fluorescence was measured. For imaging, INS-1 cells were monitored by the Laser Scanning Confocal Microscope (Zeiss), using a 506 nm laser for excitation and a 526 filter for emission. Free [Ca^2+^]_i_ were determined using a microplate reader (Molecular Devices) and the following equation: [Ca^2+^]_i_ + *K*_d_ × [F − F_min_]/F_max_ − F], where F is the fluorescence intensity of the indicator at experimental calcium levels, F_min_ is the fluorescence intensity in the absence of calcium and F_max_ is the fluorescence intensity of the calcium-saturated probe. The dissociation constant (*K*_d_) for Fluo-3 is assumed to be 325 nM. To estimate the ER Ca^2+^ concentration ([Ca^2+^]_ER_), the treated cells were loaded with Fluo-3/AM as described above and stimulated with 1 μM thapsigargin (Tg; Sigma-Aldrich). The cells were pre-incubated with EGTA (Sigma-Aldrich) prior to Tg treatment to remove the intracellular free Ca^2+^. [Ca^2+^]_ER_ was quantified by the difference between maximal [Ca^2+^]_i_ after and minimal [Ca^2+^]_i_ before Tg treatment.

### Total RNA preparation and real-time PCR

Total RNA was extracted from INS-1 cells using a High Pure RNA kit (Roche) and was reverse-transcribed with the PrimeScript™ RT-PCR Kit (Takara, Dalian, China). Real-time PCR was performed with a SYBR® Premix Ex Taq™ II kit (Takara) according to the manufacturer's instructions using Light Cycler 240 Instrument (Roche Applied Science). Relative mRNA concentrations were calculated using the 2^−ΔΔCt^ method, where Ct was the mean threshold cycle value and *36B4* was used to normalize. The primers sequences are listed in Table1.

**Table 1:** Primers sequences for real-time PCR

Gene name	Size (bp)	5′-Primer	3′-Primer	Accession number
*Atf4*	109	GTTGGTCAGTGCCTCAGACA	CATTCGAAACAGAGCATCGA	NM_024403.1
*Cat*	75	CCCGAGTCCAGGCTCTTCT	CGGCCTGTACGTAGGTGTGA	NM_012520.2
*Chop*	120	AACCTTCACTACTCTTGACCCTG	GCCATAGAACTCTGACTGGAATC	NM_001109986.1
*Dnajc3*	180	TGAAACTTGACCAGGACCAC	TGGAGCGGACTGTGTACT	NM_022232.1
*Ero1*α	169	TGAGTGAGGAGACCCAGA	CATATCCTCCAAGCGTCCG	NM_138528.1
*Gadd34*	170	GAGGGAGAAACTAAGCCAGAG	AAATCACTGTCTTCTTCCTCCTC	NM_133546.2
*Gclc*	139	TCAAGTGGGGTGACGAGG	GTTGGGTGGTTGGGGTTT	NM_012815.2
*Gclm*	189	TGTGTGATGCCACCAGATTT	GCTTTTCACGATGACCGAGT	NM_017305.2
*Gpx4*	59	CGCCGAGTGTGGTTTACGA	GCTCCTGCCTCCCGAACT	NM_001039849.1
*Grp78*	152	GAAACTGCCGAGGCGTAT	GCTGCTGTTGGCTCATTG	NM_013083.2
*Hmox1*	121	TTTTCACCTTCCCGAGCATC	GCGGTCTTAGCCTCTTCTGT	NM_012580.2
*Insulin*	166	CTACAGTCGGAAACCATCAGCA	CCACCAAGTGAGAACCACAAAG	NM_019130.2
*Ncx1*	195	CAGCACCATTGTGGGAAGCG	CAGACCTCCACGACACCAGGA	NM_001270779.1
*Nqo1*	167	GTGAGAAGAGCCCTGATTGT	CCTGTGATGTCGTTTCTGGA	NM_017000.3
*Nrf2*	145	ACATCCTTTGGAGGCAAGAC	GCCTTCTCCTGTTCCTTCTG	NM_031789.2
*PMCA2*	115	ACCTGGAAGAAGATGCCG	GCTGATTTGCTCGTGTCG	NM_012508.5
*Serca2*	122	TTTGTGGCCCGAAACTACCT	GGCATAATGAGCAGCACAAAGGG	NM_001110139.2
*Trib3*	94	GCAGAGCGGCTGATGTCT	AAGAGCAGGGCTGGTTCA	NM_144755.2
*36B4*	93	CAGCAGGTGTTTGACAATGGC	TGAGGCAACAGTCGGGTAGC	NM_022402.1

*Atf4*, activating transcription factor 4; *Cat*, catalase; *Chop*, DNA-damage inducible transcript 3; *Dnajc3*, DnaJ homologue, subfamily C, member 3; *Ero1*α, ERO1-like protein alpha; *Gadd34*, Ppp1r15a protein phosphatase 1; *Gclc*, glutamate cysteine ligase catalytic subunits; *Gclm*, glutamate cysteine ligase modulatory subunits; *Gpx4*, glutathione peroxidase 4; *Grp78*, hspa5 heat shock protein 5; *Hmox1*, haeme oxygenase (decycling) 1; *Ncx1*, sodium/calcium exchanger 1; *Nqo1*, NAD(P)H quinone oxidoreductase; *Nrf2,* nuclear factor erythroid 2-related factor 2; *PMCA2*, plasma membrane calcium-transporting ATPase 2; *Serca2*, sarcoplasmic/endoplasmic reticulum calcium ATPase 2; *Trib3*, tribbles homologue 3.

### Protein extraction and Western blot analysis

Whole-cell lysates were prepared with RIPA buffer (Boster, Wuhan, China) containing cocktails of protease and phosphatase inhibitors (Merck, Darmstadt, Germany). Cytoplasmic and nuclear fractions were separated and extracted using the NucBuster™ Protein Extraction Kit (Merck). Protein concentrations were measured using the BCA Protein Assay Kit (Thermo Scientific, Rockford, IL, USA). For Western blots, equal amounts (30 μg) of proteins were subjected to electrophoresis on SDS-PAGE followed by an electrophoretic transfer to a PVDF membrane (Bio-Rad, Hercules, CA, USA). The membrane was blocked with 5% BSA in TBST for 1.5 hrs at room temperature and incubated overnight with primary antibodies at 4°C. The blots were subsequently incubated with 1:2000 goat anti-rabbit IgG-HRP secondary antibody (Cell Signaling Technology, Beverly, MA, USA) for 1–1.5 hrs at room temperature. The antibody-reactive bands were revealed by enzyme-catalysed chemiluminescence (ECL, Beyotime Inst. Biotech) and were quantified by densitometry analysis using Image Pro Plus version 6.0 software (Media Cybernetics, Bethesda, MD, USA). Primary antibodies against insulin (sc-9168; 1:500), activating transcription factor 4 (ATF4, sc-200; 1:500) and C/EBP homologous transcription factor (CHOP, sc-575; 1:500) were purchased from Santa Cruz Biotechnology (Santa Cruz, CA, USA). Antibodies against heat shock protein 5 (GPR78, ab21685; 1:1000) were purchased from Abcam (Cambridge, UK). Antibodies against phosho-PKR-like ER kinase (p-PERK Thr980, #3179; 1:1000), PERK (#3192; 1:1000), phospho-eukaryotic initiation factor-2α (p-eIF2α Ser51, #3398; 1:1000), eIF2α (#9722; 1:1000), Caspase 3 (#9662; 1:1000), Lamin B1 (#12586; 1:1000) and β-actin (#4970; 1:2000) were purchased form Cell Signaling Technology. An antibody against Nrf2 (Q16236; 1:1000) was purchased from Epitomics.

### Statistical analyses

The data are presented as means ± SEM. Statistical analyses were performed with SPSS 17.0 software (SPSS Inc., Chicago, IL, USA) using one-way anova followed by Bonferroni's *post hoc* test. Data were considered significant when *P* < 0.05 (*), <0.01 (**) or <0.001 (***) in the comparison test.

## Results

### DEHP inhibits GSIS in INS-1 cells

**Fig 1:**
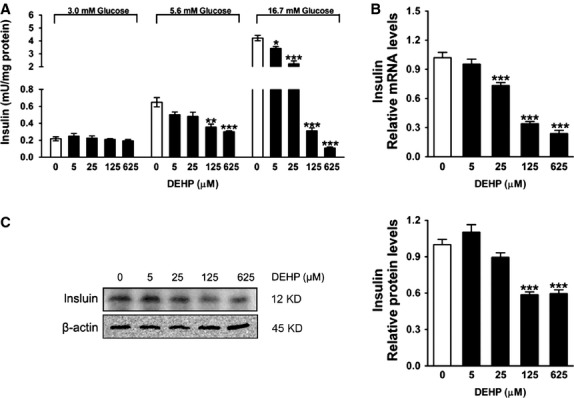
DEHP inhibits insulin secretion in INS-1 cells. (A) Glucose-stimulated insulin secretion (GSIS). Levels of secreted insulin were normalized to protein content (*n* + 4). (B) Relative mRNA amount of *insulin*. Expression levels were normalized to the housekeeping gene *36B4*. Data were collected from three independent experiments performed in triplicate. (C) Protein levels of insulin. β-actin was served as loading controls. Data were collected from three independent experiments performed in replicate. Results are expressed as mean ± SEM. *P < 0.05; **P < 0.01; ***P < 0.001 compared with untreated control cells.

Figure1A showed that insulin release at basal glucose concentration (3.0 mM) was not affected by DEHP in INS-1 cells. In addition, 5 and 25 μM DEHP did not significantly alter the insulin secretion response evoked by 5.6 mM glucose. However, insulin secretion in response to 5.6 mM glucose of the cells incubated with 125 or 625 μM DEHP for 24 hrs was reduced to 54.8% and 46.2% of untreated control cells respectively. The inhibitory effect of DEHP on insulin secretion was higher in response to 16.7 mM glucose than in response to 5.6 mM glucose. Insulin secretion in the presence of 16.7 mM glucose was decreased to 81.1, 52.8, 7.4 and 3.2% of that detected in untreated control cells after 24 hrs of incubation with 5, 25, 125 or 625 μM DEHP respectively (Fig.1A). At the molecular level, the mRNA amount of *insulin* was not affected by 5 μM DEHP in INS-1 cells, but was significantly decreased after exposure to 25, 125 or 625 μM DEHP (Fig.1B). Compared with the untreated control cells, insulin protein levels were found to be markedly decreased in the cells exposed to 125 or 625 μM DEHP (Fig.1C). No difference was detected in the level of insulin protein between 5 or 25 μM DEHP-exposed and the control cells (Fig.1C).

### DEHP decreases viability and induces apoptosis in INS-1 cells

**Fig 2:**
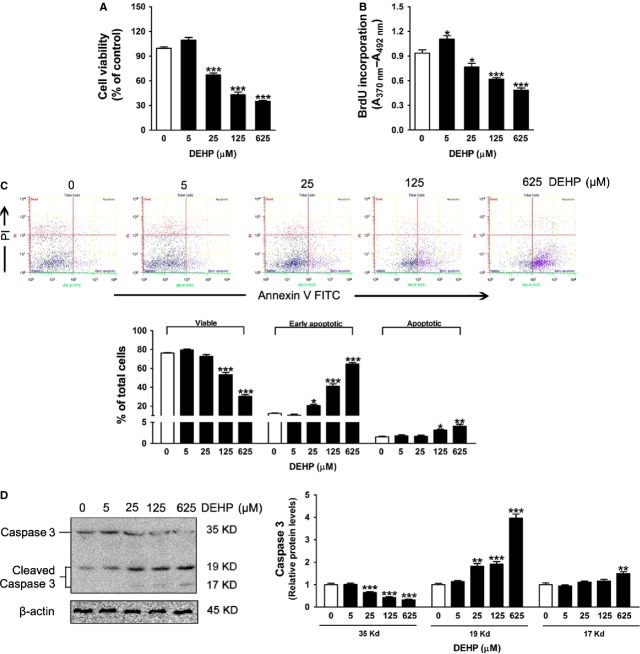
DEHP decreases cell viability and promotes apoptosis in INS-1 cells. (A) Cells viability measured by MTT assay (*n* + 6). (B) Cell proliferation measured by BrdU incorporation (*n* + 6). (C) Cells apoptosis assay using Annexin V/PI staining. The *X*-axis depicted Annexin V-positive cells and the *Y*-axis depicted PI-positive cells. Bar graphs represented the percentage of viable, early apoptotic and advanced apoptotic cells in each quadrants (*n* + 3). (D) Protein levels of caspase-3. Caspase-3 was detected as a single band at ∼35 kD and the cleaved caspase-3 was detected as doublet bands at 19 and 17 kD. β-actin was served as loading controls. Data were collected from three independent experiments performed in replicate. Results are expressed as mean ± SEM. *P < 0.05; **P < 0.01; ***P < 0.001 compared with untreated control cells.

To test the cytotoxic effect of DEHP, we initially examined the cell viability by MTT assay. Figure2A showed that cell viability was slightly higher in the 5 μM DEHP-exposed cells than that in the control, but this effect did not reach statistical significance. Conversely, DEHP treatment significantly decreased cell viability to 64.2 (25 μM), 43.3 (125 μM) or 35.1% (625 μM) of the control respectively (Fig.2A). The adverse effects of DEHP on cell viability may be related to the imbalance of cell proliferation and apoptosis. Therefore, the cell-proliferative effect was then determined by the assay using BrdU. Figure2B showed that exposure to DEHP led to changes in BrdU incorporation in a non-monotonic dose–response manner. Treatment with 25, 125 and 625 μM DEHP significantly reduced BrdU incorporation, compared with the control cells, whereas 5 μM DEHP moderately increased it (Fig.2B). To evaluate β-cell apoptosis, cells were stained with Annexin V–PI, and the results demonstrated that treatment of INS-1 cells with 25, 125 or 625 μM DEHP led to a marked and dose-dependent increase in the amount of early apoptotic cells compared with the controls cells (Fig.2C). Moreover, 125 and 625 μM DEHP also increased the amount of advanced apoptotic cells (Fig.2C). There was no difference in the extent of early apoptosis and apoptosis induction by 5 μM DEHP compared with the control (Fig.2C). Consistently, full-length caspase-3 was decreased and cleaved caspase-3 (19 kD) was increased in 25, 125 or 625 μM DEHP-exposed cells compared with controls (Fig.2D), indicating that DEHP caused caspase-3 activation and induced INS-1 cells apoptosis at higher concentrations.

### DEHP stimulates ROS production and alters Nrf2-dependent antioxidant response in INS-1 cells

**Fig 3:**
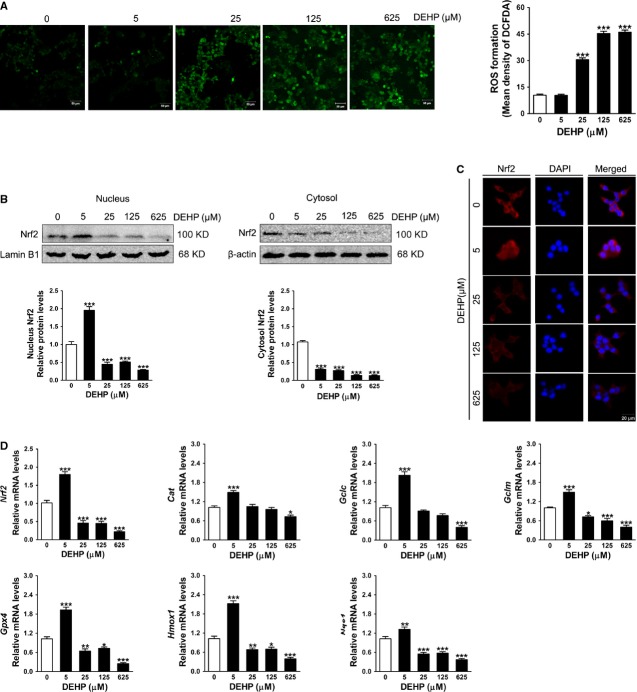
DEHP induces oxidative stress in INS-1 cells. (A) Intracellular ROS measured by DCFH-DA. The left panels showed representative images of DCFH-DA fluorescence. The bar graph showed quantitative result of images. Five images per treatment were taken: one image in each of the four quadrants and one in the centre of the well. Data were collected from five independent experiments. (B) Subcellular distribution of Nrf2 determined by Western blot analysis. Lamin B1 and β-actin were served as loading controls for the nuclear and cytosolic fractions respectively. Data were collected from three independent experiments performed in replicate. (C) Representative images of intracellular localization of Nrf2 determined by immunofluorescence (400× magnification). Nucleus was stained with DAPI (blue) and Nrf2 was probed with a primary anti-Nrf2 antibody (red). The merging of Nrf2 and DAPI was also shown. (D) Relative mRNA amount of *Nrf2* and its target genes. Expression levels were normalized to the housekeeping gene *36B4*. Data were collected from three independent experiments performed in triplicate. Results are expressed as mean ± SEM. *P < 0.05; **P < 0.01; ***P < 0.001 compared with untreated control cells.

Intracellular ROS levels were measured using a DCFH-DA probe and the results showed that DCFH-DA fluorescence was increased significantly in the cells exposed to 25, 125 or 625 μM DEHP compared with controls (Fig.3A). As excess ROS generation may be related to an inadequate of antioxidant defence response, we next measured the nuclear accumulation of Nrf2 as well as the transcription of Nrf2 and its target genes. Figure3B–D showed that higher concentrations of DEHP significantly inhibited the Nrf2-dependent antioxidant response. Both the protein levels of Nrf2 in the nucleus and the cytosol and the mRNA amounts of *Nrf2* and its downstream antioxidant enzyme genes, *Nqo1*, *Gclm*, *Hmox1* and *Gpx4*, were significantly decreased in the cells exposed to 25, 125 or 625 μM DEHP when compared with the control. In addition, mRNA of the *Cat* and *Gclc* were also decreased after exposure to 625 μM DEHP (Fig.3D). In contrast, 5 μM DEHP activated the Nrf2-mediated adaptive response in INS-1 cells. Figure3B showed that the nuclear Nrf2 was increased but cytosolic Nrf2 was decreased in the 5 μM DEHP-exposed cells compared with the control. Similar results were observed in immunofluorescence analysis of Nrf2 localization, showing that 5 μM DEHP treatment slightly increased perinuclear localization and nuclear translocation of Nrf2 (Fig.3C). Apart from nuclear translocation, 5 μM DEHP also induced transcriptional up-regulation of Nrf2 and many Nrf2-target genes such as *Nqo1*, *Gclc*, *Gclm*, *Cat*, *Hmox1* and *Gpx4* in INS-1 cells (Fig.3D).

### DEHP induces the ER stress response in INS-1 cells

**Fig 4:**
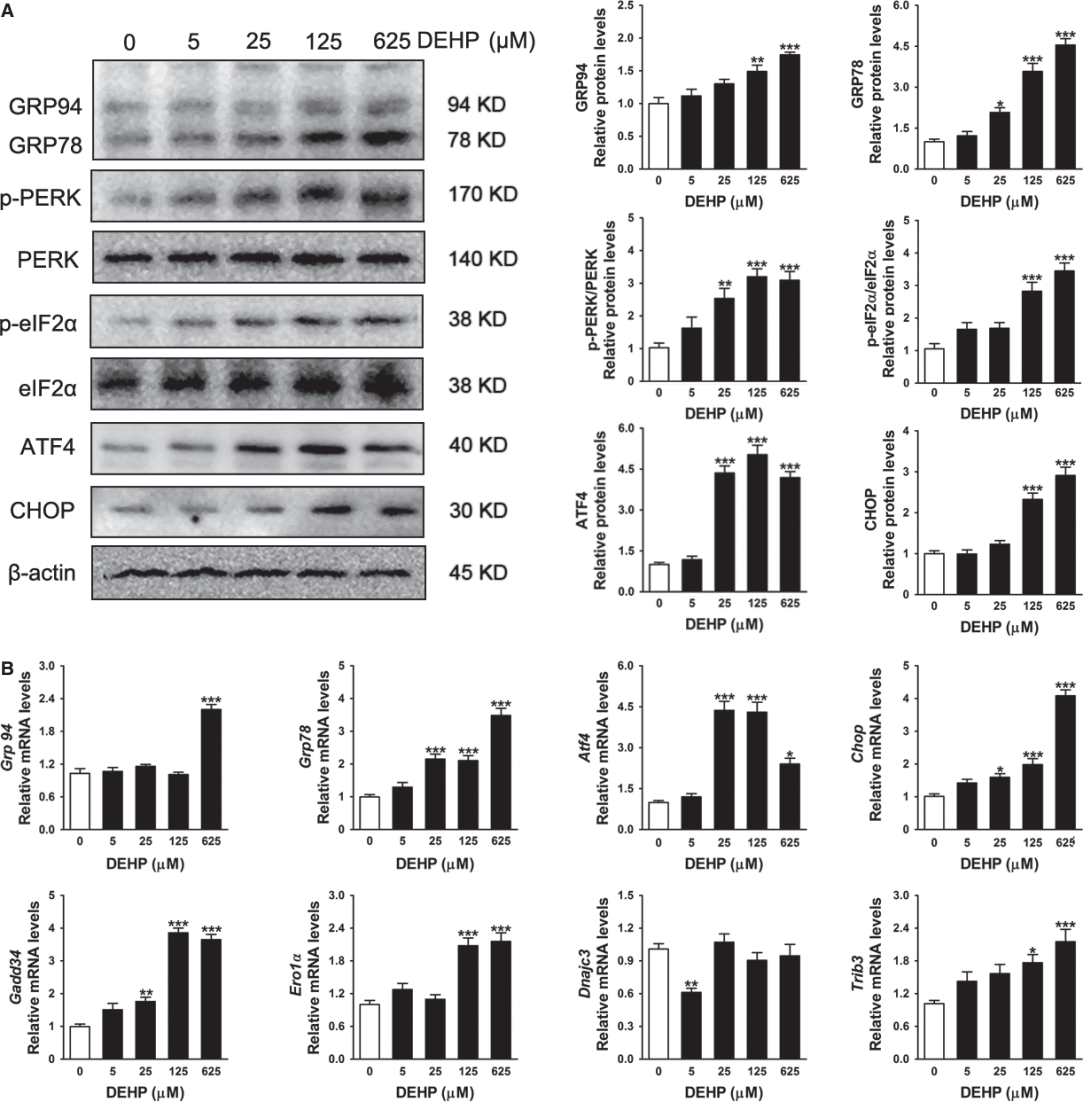
DEHP activates ER stress response in INS-1 cells. (A) Protein levels of PERK–ATF4–CHOP ER stress signalling pathway. β-actin was served as loading controls. Data were collected from three independent experiments performed in replicate. (B) Relative mRNA amount of genes involved in ER stress. Expression levels were normalized to the housekeeping gene *36B4*. Data were collected from three independent experiments performed in triplicate. Results are expressed as mean ± SEM. *P < 0.05; **P < 0.01; ***P < 0.001 compared with untreated control cells.

Endoplasmic reticulum stress pathway signalling and the levels of downstream target genes were measured to test the hypothesis that early apoptosis occurred in INS-1 cells treated with DEHP as a result of ER stress response. Figure4 showed that both the mRNA and protein levels of an ER chaperones, GRP78 were dose-dependently up-regulated in cells exposed to 25, 125 or 625 μM DEHP. Similarly, the level of GRP94 protein was significantly increased in 25 or 625 μM DEHP-exposed cells and its mRNA levels were increased by exposure to 625 μM DEHP. As GRP94 binds to the luminal domains of the ER stress transducer PERK, activation of the PERK pathways was examined next. Figure4A showed that the amounts of phosphorylation of PERK and its substrate eIF2α were significantly increased after exposure to 25, 125 or 625 μM DEHP. Phosphorylated PERK and eIF2α were also slightly elevated in cells exposed to 5 μM DEHP; however, the effects did not reach statistical significance (Fig.4A). Levels of ATF4 mRNA and protein, known to be induced by eIF2α phosphorylation, were significantly elevated in the cells after exposure to 25, 125 or 625 μM DEHP (Fig.4). Apart from eliciting the ER stress signalling pathways, 125 or 625 μM DEHP dramatically increased transcription and translation of a pro-apoptotic factors, CHOP (Fig.4). mRNA levels of several target genes of CHOP that encode pro-apoptotic functions, such as *Gadd34* and *Trib3*, were also up-regulated in the cells exposed to 125 or 625 μM DEHP compared with the control (Fig.4B). The amount of CHOP-dependent *Ero1*α, which can generate oxidizing equivalents in the ER, was shown to be induced by 125 or 625 μM DEHP in INS cells (Fig.4B). In addition, mRNA level of *Dnajc3* was decreased in 5 μM DEHP-exposed cells, but unaltered in 25, 125 or 625 μM DEHP-exposed cells when compared with untreated controls (Fig.4B).

### DEHP disturbs Ca^2+^ homoeostasis in INS-1 cells

**Fig 5:**
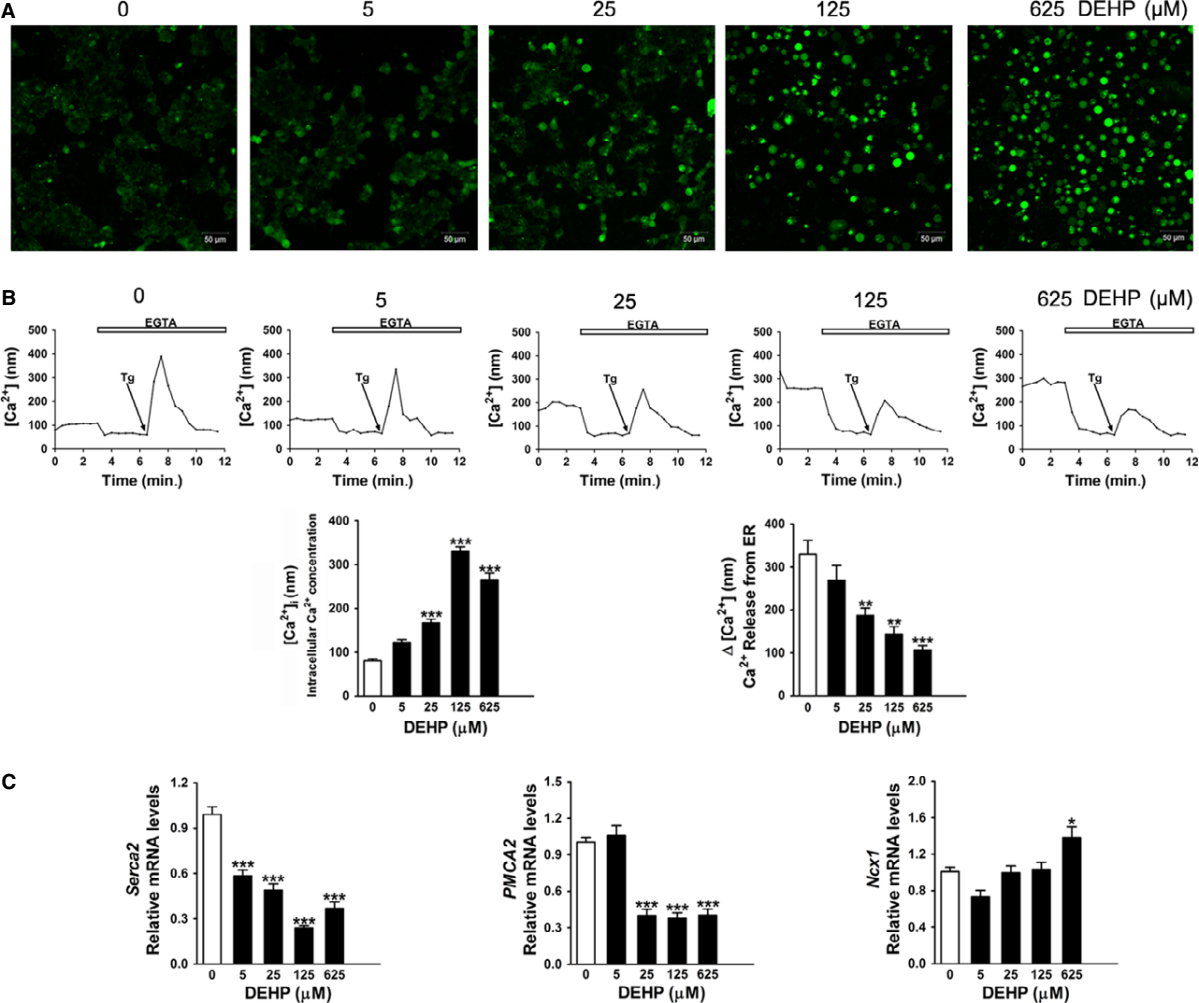
DEHP disturbs Ca^2+^ homoeostasis in INS-1 cells. (A) Representative images of Ca^2+^ in cells stained with Fluo-3/AM at 200× magnification. (B) Quantification of intracellular free Ca^2+^ concentration ([Ca^2+^]_i_) and ER Ca^2+^ concentration ([Ca^2+^]_ER_). [Ca^2+^]_i_ was measured at baseline prior to EGTA treatment. [Ca^2+^]_ER_ was determined by the Tg-mediated [Ca^2+^]_i_ increase (Δ[Ca^2+^]). Δ[Ca^2+^] was quantified by the difference between maximal [Ca^2+^]_i_ after and minimal [Ca^2+^]_i_ before Tg treatment. Cells were pre-treated with 4 mM EGTA to reduce cellular background Ca^2+^ prior to treatment with Tg. The traces shown were the means of four independent experiments. (C) Relative mRNA amount of *Serca2*, *PMCA2* and *Ncx1*. Expression levels were normalized to the housekeeping gene *36B4*. Data were collected from three independent experiments performed in triplicate. Results are expressed as mean ± SEM. *P < 0.05; **P < 0.01; ***P < 0.001 compared with untreated control cells.

Because aberrant Ca^2+^ homoeostasis has been shown to mediate ER stress and β-cell apoptosis, the effect of DEHP on Ca^2+^ homoeostasis was evaluated with a fluorescent Ca^2+^ indicator Fluo-3/AM. Figure5A showed that DEHP exposure led to a significant and dose-dependent increase in [Ca^2+^]_i_ in INS-1 cells. Subsequently, cells were treated with Tg, which was able to dissipate ER Ca^2+^ storage resulting in increased cytosolic free Ca^2+^ which indirectly reflected the Ca^2+^ level at ER 23. As shown in Figure5B, untreated control cells exhibited a sharp increase in [Ca^2+^]_i_ response to Tg stimulation, but Tg-driven increase in [Ca^2+^]_i_ was dose-dependently blunted when cells were exposed to DEHP. Likewise, exposure to 5–625 μM DEHP significantly reduced the mRNA levels of *Serca2*, which is essential for the maintenance of calcium movement across the cells. The expression of *PMCA2* which encodes a major Ca^2+^ extrusion pump involved in regulation of Ca^2+^ signalling, were also reduced in cells exposed to 25, 125 or 625 μM DEHP. In contrast, 625 μM DEHP induced an increase in the mRNA level of Na/Ca Exchanger *Ncx1*.

**Fig 6:**
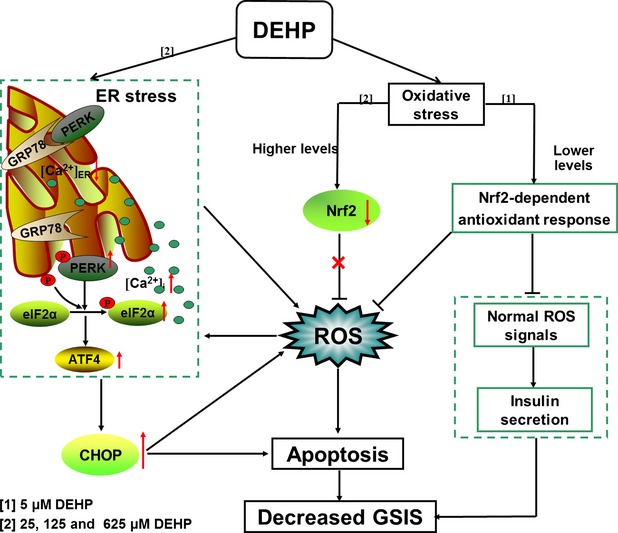
Schematic diagram of the signalling pathways involved in DEHP-induced β-cell dysfunction. We showed that exposure to 25, 125 or 625 μM DEHP induced persistent ER stress and significant oxidative damage in INS-1 cells, ultimately contributing to cell dysfunction and apoptosis. DEHP caused ER stress in INS-1 cells mainly by elevating transcription and translation of ER chaperone, activation of PERK–ATF4–CHOP signalling pathway, as well as depletion of ER Ca^2+^. Apart from ER stress, DEHP triggered oxidative stress in INS-1 cells. However, cellular defence differed between cells treated with low and high levels of DEHP. Of DEHP, 5 μM activated Nrf2-mediated antioxidant response, but it also blunted physiological ROS signalling, thereby reducing insulin secretion in INS-1 cells. Of DEHP, 25, 125 or 625 μM degraded Nrf2, blocked the Nrf2-dependent antioxidant defence protection and further increased the production of ROS in INS-1 cells, which resulted in irreversible oxidative damage and cell apoptosis.

## Discussion

Exposure of humans to DEHP happens quite frequently and has been associated with the occurrence of type 2 diabetes. However, the underlying mechanisms mediating these adverse effects remain poorly understood. Considering that pancreatic β cells play a pivotal role in the maintenance of glucose homoeostasis, the present study was designed to investigate the cytotoxic effects of DEHP on the insulinoma-derived β cells INS-1, and explore the associated molecular mechanisms. INS-1 cells generated by Asfari *et al*. in 1992 display many important characteristics of the native β cells, including a high insulin content and responsiveness to glucose stimulation 24. However, some discrepancies were observed among numerous laboratories using INS-1 cells, which might be attributed to the non-clonal nature of INS-1 cells. To circumvent this problem, Claes Wolheim *et al*. isolated clonal INS-1E cells from parental INS-1 cells. INS-1E cells displayed stable differentiated β cell phenotype more than 100 passages and were able to secrete insulin in response to elevated glucose concentrations 25,26. Unfortunately, INS-1E cells are not commercially available. Asfari *et al*. indicated that the amount of insulin in INS-1 cells remained unchanged over 80 passages. Moreover, INS-1 cells are highly susceptible to damage under oxidative stress because of their low expression of oxygen radical scavenging enzymes, mimicking the susceptibility of primary islets. Therefore, INS-1 cells have now been extensively used for the study of oxidative stress-mediated dysfunction and cytotoxicity 27. In the present study, we demonstrated that DEHP exposure significantly and dose-dependently decreased insulin secretion ability and induced early apoptosis in INS-1 cells. More importantly, we provided evidence that the adverse effects of DEHP on INS-1 cells were mediated by the activation of PERK–ATF4–CHOP ER stress signalling pathway and the dysregulations of Nrf2-dependent antioxidant protection.

DEHP is widely dispersed in the environment. As one example, a recent study of monitoring DEHP in 623 food samples found DEHP in almost all of the tested samples, including milk-based products, distilled liquor, wine, beverages, grains, meat, oil, biscuits (cookies) and canned food, with levels ranging from 0.02 to 2685 mg/kg 28. Another representative survey of phthalates in U.S. food also indicated that DEHP concentrations were the highest of the phthalates measured in all foods (except beef) and pork contained the highest concentration of DEHP (mean 300 mg/kg; max 1158 mg/kg) 29. In addition to the ingestion of contaminated food, individuals can be exposed to DEHP *via* inhalation of polluted air and dermal contact with tainted products. DEHP exposure in the general population from all sources is estimated to be 3–30 μg/kg/day 30. Occupational exposure and specific medical treatments using PVC medical devices lead to DEHP exposure levels that are substantially higher than background levels. For example, the amount of DEHP in blood products stored in PVC bags ranged from 1.8 to 83.2 μg/ml (0.5–213.0 μM) and the amount would increase with increasing storage time 31. Long-term haemodialysis resulted in the highest cumulative dose of DEHP (up to 2.2 mg/kg/day) in adults, and short-term blood transfusions to trauma patients also led to the highest acute DEHP exposure (up to 10 mg/kg/day) 32. Moreover, almost all neonates in medical care have been shown to be highly exposed to DEHP. Maximum DEHP exposure of neonates exceeded the reference dose (20 μg/kg/day) and the tolerable daily intake (50 μg/kg/day) by a factor of 100 33. DEHP absorbed in the body could be metabolized into MEHP; therefore, there were studies using MEHP to evaluate the effects of DEHP *in vitro*. Nevertheless, another study suggested that DEHP is active at the cellular level because it has some intrinsic activity in mediating cytotoxic effects. Moreover, cells have a capacity to convert of DEHP to its metabolites 34. Studies *in vitro* also indicated that MEHP cannot pass the plasma membrane and enter into cells as readily as DEHP can 35. Most importantly, it has been reported that ∼7.3% of DEHP is metabolized to MEHP and 25.8% of DEHP remains accumulated in body tissues after it entered into the human body 36. In this study, we focused on the direct action of DEHP on INS-1 cells and we chose concentration ranges ranging from 5 to 625 μM based on experimental design of many *in vitro* studies 34,37,38.

Oxidative stress is widely implicated as a pathogenetic mechanism of DEHP toxicity. Numerous studies have confirmed that DEHP induces reproductive toxicity mainly by inducing ROS production and by disrupting the activity of antioxidant enzymes 16,17,34. In hepatocytes, DEHP has also been reported to induce apoptosis *via* the activation of the ERK/NF-κB signalling pathway, in which intracellular Ca^2+^ and ROS act as pivotal mediators 37. Consistent with these studies, our study revealed that ROS generation was significantly increased in INS-1 cells exposed to 25, 125 or 625 μM DEHP. In addition, we found that DEHP at these concentrations decreased the amount of Nrf2 in both the nucleus and the cytosol, with a corresponding decrease in the transcription of the genes encoding antioxidant enzymes, such as *Hmox-1*, *Cat*, *Gclc*, *Gclm*, *Nqo1* and *GPx*. Nrf2 is one of the most important cellular defence mechanisms that can neutralize ROS and detoxify harmful chemicals to limit oxidative damage and maintain cellular redox homoeostasis 39,40. Blunting the Nrf2-mediated antioxidant response would result in irreversible oxidative cellular damage. Indeed, insulin secretion was decreased and cellular apoptosis was demonstrated by positive Annexin–PI staining and caspase-3 activation in a dose-dependent manner in DEHP-exposed INS-1 cells. Surprisingly, 5 μM DEHP decreased GSIS in INS-1 cells, but did not increase ROS production, implying that low and high DEHP doses might work through different mechanisms to impair insulin secretion. Under lower dose DEHP stimulation, the nuclear translocation and accumulation of Nrf2 and increase transcription of Nrf2 target genes were exhibited, indicating that INS-1 cells are able to trigger a defence mechanism to counteract low-level DEHP toxicity. Although the activation of the Nrf2-mediated antioxidant response has the potential to detoxify ROS and alleviate oxidative damage, it can also block normal glucose-dependent ROS signals involving in insulin secretion 41. In this situation, Nrf2 activation might become the primary cause of decreased GSIS observed in the cells exposed to 5 μM DEHP. Collectively, these data indicated a critical role for the Nrf2-mediated antioxidant response in DEHP-induced β-cell dysfunction and death.

Endoplasmic reticulum is one of major sites in cells for protein synthesis and ER stress is currently considered a crucial event that drives cell apoptosis. β cells possess an extremely well-developed ER because of their functions in secreting insulin and glycoproteins; they are susceptible to changes in ER homoeostasis. ROS is reported to be an important inducer of ER stress, and ER stress in turn exacerbates the accumulation of excess ROS, ultimately entering a vicious circle and leading to cell dysfunction and apoptosis. In this study, we first found that exposure to DEHP significantly and dose-dependently induced ROS generation in INS-1 cells. Therefore, whether exposure to DEHP would trigger the ER stress response was analysed in subsequent experiments. It is known that the ER proteins PERK, ATF6 and IRE-1 are maintained in an inactive state by binding with ER-localized chaperones GRP78 and GRP94. ER stress can cause activation of PERK, ATF6 and IRE-1, as well as their downstream signalling pathways. This study focused only on the PERK–eIF2α signalling pathway because the PERK branch is the only pathway in ER stress that has been shown to induce an antioxidant stress response 42,43. As expected, we found that a 24-hr DEHP treatment elicited ER stress in INS-1 cells, as demonstrated by the up-regulation of the major ER-localized chaperones GRP78 and GRP94, as well as an increase in PERK phosphorylation concomitant with the stimulation of eIF2α phosphorylation. Simultaneous to the ER stress response, there was a significant decrease in insulin content and secretion in INS-1 cells after exposure to DEHP, demonstrating that ER stress partially contributed to β-cell dysfunction.

PERK is able to co-ordinate the convergence of ER stress with oxidative stress signalling, resulting in Nrf2 and ATF4 activation 44. However, the data presented in this study suggested that DEHP-mediated activation of PERK demonstrated no obvious effect on the nuclear translocation of Nrf2 and the subsequent induction of target genes induction in INS-1 cells. Both nuclear and cytoplasmic Nrf2 were decreased in INS-1 cells when they were exposed to 25, 125 or 625 μM DEHP, despite that phosphorylation of PERK was increased in these cells. The decline of Nrf2 levels and activity could be consequences of the large amounts of ROS-mediated cellular damage. On the other hand, PERK-dependent phosphorylation of eIF2α unexpectedly enhanced the transcription and translation levels of ATF4 and CHOP in INS-1 cells after DEHP treatment. CHOP is considered to be a crucial regulator of ER stress-related apoptosis signalling 45. CHOP mediates cell apoptosis through the induction of genes such as *Gadd34* and *Ero1*α 46. *Ero1*α contributes significantly to the accumulation and production of ROS in ER stressed cells 43, and so the observations made here suggested that 125 and 625 μM DEHP could facilitate ROS formation inside the ER by activating *Ero1*α, finally leading to apoptosis in INS-1 cells. Of note, activation of *Ero1*α-derived ROS is mainly confined to the ER, while the Nrf-2-mediated antioxidant response is triggered by ROS accumulation in the cytosol 47, which could partially explain why higher doses of DEHP did not activate Nrf2 in INS-1 cells. Although an up-regulation of *Gadd34* expression was also observed in INS-1 cells exposed to high levels of DEHP, the negative feed-back control of Gadd34-mediated p-eIF2a dephosphorylation 48 was not observed, suggesting that DEHP-exposed INS-1 cells might possess a specific resistance to p-eIF2α dephoshporylation, but this remained to be elucidated in the future studies. In addition to *Gadd34* and *Ero1*α, higher level DEHP increased transcription of *Trib3*, a novel ER stress-inducible gene and a target of CHOP/ATF4 involved in ATF4/CHOP-mediated apoptosis as a second messenger during ER stress response 49,50. The balance between pro-apoptotic and anti-apoptotic processes determines the fate of β cells. Increased apoptosis would result in a reduction in β-cell number and loss of mass, finally leading to a deterioration of key β-cell functions, such as GSIS. Therefore, we suggested that DEHP-mediated activation of some apoptotic signals, such as increases in the expression of *Trib3* in β cells, might specifically blunt insulin signalling and inhibit the GSIS response in INS-1 cells.

Endoplasmic reticulum is a major intracellular source for Ca^2+^. The maintenance of Ca^2+^ homoeostasis plays a key role in several aspects of β-cell physiology, including insulin production and secretion and the maintenance of ER function. The ER stress response can be activated in conditions of ER Ca^2+^ depletion. After establishing that DEHP triggered the ER stress response in INS-1 cells, this study subsequently investigated the possibility that DEHP would perturb the normal regulation of ER Ca^2+^ homoeostasis. Our data showed that DEHP significantly decreased ER Ca^2+^ storage and increased the intracellular free Ca^2+^ ([Ca^2+^]_i_), paralleling the activation of ER stress. Elevated [Ca^2+^]_i_ observed in DEHP-exposed cells was likely a result of enhancing Ca^2+^ leakage from the ER. In this study, we showed that DEHP exposure significantly decreased the expression of *Serca2b*, one of the predominant SERCA isoforms expressed in the pancreatic islet, which was consistent with the data from the studies of rodent diabetic islets 51,52 and human islets isolated from cadaveric T2DM diabetic donors 53. Because *Serca2b* is mainly responsible for removing Ca^2+^ from the cytosol back into ER Ca^2+^ storage 54, DEHP-induced down-regulation of *Serca2b* expression could result in an unbalance between the continuous leakage of Ca^2+^ from the ER and the SERCA pump mediated reuptake of Ca^2+^, ultimately causing ER Ca^2+^ depletion and activation of the ER stress. In addition, *PMCA2* transcription levels were decreased by the treatment with 25, 125 or 625 μM DEHP, whereas the amount of *Ncx1* was increased with 625 μM DEHP. PCAM and NCX are responsible for Ca^2+^ extrusion and clearance from the β cells 55,56. The changes in *PMCA2* and *Ncx1* levels would result in an inability to transport the increased Ca^2+^ load, the progressive intracellular Ca^2+^ toxicity, and ultimately, activation of apoptosis in INS-1 cells after higher doses of DEHP.

In conclusion, the present study provided the first evidence that oxidative stress and ER stress response were able to be induced by DEHP in INS-1 cells, where they may interrelate and form a ‘stress loop’, finally contributing to the abrogated GSIS, reduced insulin content and the activation of apoptosis. DEHP is an important environmental risk factor for type 2 diabetes. However, we acknowledge that there are some limitations of this study: there are difficulties in extrapolating the result obtained *in vitro* to that *in vivo*; therefore, this study cannot be taken as clear evidence for concern regarding real-life human exposure. To address this question, further analysis in animal models are necessary to better understand the cellular and molecular mechanisms of DEHP on pancreatic β cells on the basis of the observed effects of this study.

## References

[b1] Kelley KE, Hernández-Díaz S, Chaplin EL (2012). Identification of phthalates in medications and dietary supplement formulations in the United States and Canada. Environ Health Perspect.

[b2] Schettler T (2006). Human exposure to phthalates *via* consumer products. Int J Androl.

[b3] CDC (2012). http://www.cdc.gov/exposurereport/pdf/FourthReport_UpdatedTables_Sep2012.pdf.

[b4] Saravanabhavan G, Guay M, Langlois É (2013). Biomonitoring of phthalate metabolites in the Canadian population through the Canadian Health Measures Survey (2007–2009). Int J Hyg Environ Health.

[b5] Stahlhut RW, van Wijngaarden E, Dye TD (2007). Concentrations of urinary phthalate metabolites are associated with increased waist circumference and insulin resistance in adult US males. Environ Health Perspect.

[b6] Svensson K, Hernández-Ramírez RU, Burguete-García A (2011). Phthalate exposure associated with self-reported diabetes among Mexican women. Environ Res.

[b7] James-Todd T, Stahlhut R, Meeker JD (2012). Urinary phthalate metabolite concentrations and diabetes among women in the National Health and Nutrition Examination Survey (NHANES) 2001–2008. Environ Health Perspect.

[b8] Selvaraj J, Balasubramanian K, Pei F (2013). Phthalate is associated with insulin resistance in adipose tissue of male rat: role of antioxidant vitamins. J Cell Biochem.

[b9] Srinivasan C, Khan AI, Balaji V (2011). Diethyl hexyl phthalate-induced changes in insulin signaling molecules and the protective role of antioxidant vitamins in gastrocnemius muscle of adult male rat. Toxicol Appl Pharmacol.

[b10] Butler AE, Janson J, Bonner-Weir S (2003). β-Cell deficit and increased β-cell apoptosis in humans with type 2 diabetes. Diabetes.

[b11] Lenzen S (2008). Oxidative stress: the vulnerable beta-cell. Biochem Soc Trans.

[b12] Lenzen S, Drinkgern J, Tiedge M (1996). Low antioxidant enzyme gene expression in pancreatic islets compared with various other mouse tissues. Free Radical Biol Med.

[b13] Dzhekova-Stojkova S, Bogdanska J, Stojkova Z (2001). Peroxisome proliferators: their biological and toxicological effects. Clin Chem Lab Med.

[b14] Ferguson KK, Loch-Caruso R, Meeker JD (2012). Exploration of oxidative stress and inflammatory markers in relation to urinary phthalate metabolites: NHANES 1999-2006. Environ Sci Technol.

[b15] Giral P, Ratziu V, Couvert P (2010). Plasma bilirubin and gamma-glutamyltransferase activity are inversely related in dyslipidemic patients with metabolic syndrome: relevance to oxidative stress. Atherosclerosis.

[b16] Erkekoglu P, Rachidi W, Yuzugullu OG (2010). Evaluation of cytotoxicity and oxidative DNA damaging effects of di (2-ethylhexyl)-phthalate (DEHP) and mono (2-ethylhexyl)-phthalate (MEHP) on MA-10 Leydig cells and protection by selenium. Toxicol Appl Pharmacol.

[b17] Wang W, Craig ZR, Basavarajappa MS (2012). Di (2-ethylhexyl) phthalate inhibits growth of mouse ovarian antral follicles through an oxidative stress pathway. Toxicol Appl Pharmacol.

[b18] Bhandary B, Marahatta A, Kim H-R (2012). An involvement of oxidative stress in endoplasmic reticulum stress and its associated diseases. Int J Mol Sci.

[b19] Guan L, Han B, Li Z (2009). Sodium selenite induces apoptosis by ROS-mediated endoplasmic reticulum stress and mitochondrial dysfunction in human acute promyelocytic leukemia NB4 cells. Apoptosis.

[b20] Fonseca SG, Gromada J, Urano F (2011). Endoplasmic reticulum stress and pancreatic β-cell death. Trends Endocrinol Metab.

[b21] Lin Y, Wei J, Li Y (2011). Developmental exposure to di (2-ethylhexyl) phthalate impairs endocrine pancreas and leads to long-term adverse effects on glucose homeostasis in the rat. Am J Physiol Endocrinol Metabol.

[b22] Tetz LM, Cheng A, Korte C (2013). Mono-2-ethylhexyl phthalate induces oxidative stress responses in human placental cells *in vitro*. Toxicol Appl Pharmacol.

[b23] Lytton J, Westlin M, Hanley MR (1991). Thapsigargin inhibits the sarcoplasmic or endoplasmic reticulum Ca-ATPase family of calcium pumps. J Biol Chem.

[b24] Asfari M, Janjic D, Meda P (1992). Establishment of 2-mercaptoethanol-dependent differentiated insulin-secreting cell lines. Endocrinology.

[b25] Merglen A, Theander S, Rubi B (2004). Glucose sensitivity and metabolism-secretion coupling studied during two-year continuous culture in INS-1E insulinoma cells. Endocrinology.

[b26] Janjic D, Maechler P, Sekine N (1999). Free radical modulation of insulin release in INS-1 cells exposed to alloxan. Biochem Pharmacol.

[b27] Hohmeier HE, Newgard CB (2004). Cell lines derived from pancreatic islets. Mol Cell Endocrinol.

[b28] Xu D, Deng X, Fang E (2014). Determination of 23 phthalic acid esters in food by liquid chromatography tandem mass spectrometry. J Chromatogr.

[b29] Schecter A, Lorber M, Guo Y (2013). Phthalate concentrations and dietary exposure from food purchased in New York State. Environ Health Perspect.

[b30] NTP-CERHR (2003). http://cerhr.niehs.gov/news/index.html.

[b31] Inoue K, Kawaguchi M, Yamanaka R (2005). Evaluation and analysis of exposure levels of di (2-ethylhexyl) phthalate from blood bags. Clin Chim Acta.

[b32] Commission E (2008).

[b33] Koch HM, Preuss R, Angerer JD (2006). Di (2-ethylhexyl) phthalate (DEHP): human metabolism and internal exposure–an update and latest results1. Int J Androl.

[b34] Ambruosi B, Uranio MF, Sardanelli AM (2011). *In vitro* acute exposure to DEHP affects oocyte meiotic maturation, energy and oxidative stress parameters in a large animal model. PLoS ONE.

[b35] Kristensen DM, Skalkam ML, Audouze K (2011). Many putative endocrine disruptors inhibit prostaglandin synthesis. Environ Health Perspect.

[b36] Wittassek M, Angerer J (2008). Phthalates: metabolism and exposure. Int J Androl.

[b37] Ghosh J, Das J, Manna P (2010). Hepatotoxicity of di-(2-ethylhexyl) phthalate is attributed to calcium aggravation, ROS-mediated mitochondrial depolarization, and ERK/NF-κB pathway activation. Free Radical Biol Med.

[b38] Chen X, Qin Q, Zhang W (2013). Activation of the PI3K-AKT-mTOR signaling pathway promotes DEHP-induced Hep3B cell proliferation. Food Chem Toxicol.

[b39] Zhang DD (2006). Mechanistic studies of the Nrf2-Keap1 signaling pathway*. Drug Metab Rev.

[b40] Kensler TW, Wakabayashi N, Biswal S (2007). Cell survival responses to environmental stresses *via* the Keap1-Nrf2-ARE pathway. Annu Rev Pharmacol Toxicol.

[b41] Pi J, Zhang Q, Fu J (2010). ROS signaling, oxidative stress and Nrf2 in pancreatic beta-cell function. Toxicol Appl Pharmacol.

[b42] Hansen HG, Schmidt JD, Søltoft CL (2012). Hyperactivity of the Ero1α oxidase elicits endoplasmic reticulum stress but no broad antioxidant response. J Biol Chem.

[b43] Harding HP, Zhang Y, Zeng H (2003). An integrated stress response regulates amino acid metabolism and resistance to oxidative stress. Mol Cell.

[b44] Cullinan SB, Diehl JA (2006). Coordination of ER and oxidative stress signaling: the PERK/Nrf2 signaling pathway. Int J Biochem Cell Biol.

[b45] Zinszner H, Kuroda M, Wang X (1998). CHOP is implicated in programmed cell death in response to impaired function of the endoplasmic reticulum. Genes Dev.

[b46] Marciniak SJ, Yun CY, Oyadomari S (2004). CHOP induces death by promoting protein synthesis and oxidation in the stressed endoplasmic reticulum. Genes Dev.

[b47] Itoh K, Mimura J, Yamamoto M (2010). Discovery of the negative regulator of Nrf2, Keap1: a historical overview. Antioxid Redox Signal.

[b48] Novoa I, Zeng H, Harding HP (2001). Feedback inhibition of the unfolded protein response by GADD34-mediated dephosphorylation of eIF2α. J Cell Biol.

[b49] Morse E, Schroth J, You Y-H (2010). TRB3 is stimulated in diabetic kidneys, regulated by the ER stress marker CHOP, and is a suppressor of podocyte MCP-1. Am J Physiol Renal Physiol.

[b50] Qian B, Wang H, Men X (2008). TRIB3 is implicated in glucotoxicity-and endoplasmic reticulum-stress-induced beta-cell apoptosis. J Endocrinol.

[b51] Roe MW, Philipson LH, Frangakis CJ (1994). Defective glucose-dependent endoplasmic reticulum Ca^2+^ sequestration in diabetic mouse islets of Langerhans. J Biol Chem.

[b52] Cardozo AK, Ortis F, Storling J (2005). Cytokines downregulate the sarcoendoplasmic reticulum pump Ca^2+^ ATPase 2b and deplete endoplasmic reticulum Ca^2+^, leading to induction of endoplasmic reticulum stress in pancreatic β-cells. Diabetes.

[b53] Kono T, Ahn G, Moss DR (2012). PPAR-γ activation restores pancreatic islet SERCA2 levels and prevents β-cell dysfunction under conditions of hyperglycemic and cytokine stress. Mol Endocrinol.

[b54] Arredouani A, Guiot Y, Jonas J-C (2002). SERCA3 ablation does not impair insulin secretion but suggests distinct roles of different sarcoendoplasmic reticulum Ca^2+^ pumps for Ca^2+^ homeostasis in pancreatic β-cells. Diabetes.

[b55] Carafoli E (1988). Membrane transport of calcium: an overview. Methods Enzymol.

[b56] Blaustein MP, Lederer WJ (1999). Sodium/calcium exchange: its physiological implications. Physiol Rev.

